# Controlled functional expression of the bacteriocins pediocin PA-1 and bactofencin A in *Escherichia coli*

**DOI:** 10.1038/s41598-017-02868-w

**Published:** 2017-06-08

**Authors:** Beatriz Mesa-Pereira, Paula M. O’Connor, Mary C. Rea, Paul D. Cotter, Colin Hill, R. Paul Ross

**Affiliations:** 1 Teagasc Food Research Centre, Teagasc Moorepark, Fermoy, Co., Cork, Ireland; 2 APC Microbiome Institute, University College Cork, Cork, Ireland; 3 Department of Microbiology, University College Cork, Cork, Ireland

## Abstract

The bacteriocins bactofencin A (class IId) and pediocin PA-1 (class IIa) are encoded by operons with a similarly clustered gene organization including a structural peptide, an immunity protein, an ABC transporter and accessory bacteriocin transporter protein. Cloning of these operons in *E*. *coli* Tuner^TM^ (DE3) on a pETcoco-2 derived vector resulted in successful secretion of both bacteriocins. A corresponding approach, involving the construction of vectors containing different combinations of these genes, revealed that the structural and the transporter genes alone are sufficient to permit heterologous production and secretion in this host. Even though the accessory protein, usually associated with optimal disulfide bond formation, was not required for bacteriocin synthesis, its presence did result in greater pediocin PA-1 production. The simplicity of the system and the fact that the associated bacteriocins could be recovered from the extracellular medium provides an opportunity to facilitate protein engineering and the overproduction of biologically-active bacteriocins at industrial scale. Additionally, this system could enable the characterization of new bacteriocin operons where genetic tools are not available for the native producers.

## Introduction

Antimicrobial peptides (AMPs) are produced by numerous organisms including many Gram-positive bacteria as a part of their innate defenses^1, 2^. AMPs secreted by *Lactococcus*, *Lactobacillus*, *Pediococcus* and other genera of lactic acid bacteria (LAB), have attracted considerable attention due to their activity against spoilage and pathogenic bacteria^3^. Several of these peptides have proven applications as natural preservatives in the food industry or have potential as alternatives to antibiotics in veterinary and medical applications^4–6^. These bacterial AMPs, also called bacteriocins, comprise a heterogeneous family of gene-encoded, small ribosomally synthesized peptides with a narrow or broad inhibitory spectrum^2, 7^.

According to Cotter *et al*.^4^, bacteriocins are classified into those which are either post-translationally modified (class I) and unmodified or minimally modified peptides (class II)^2, 5^. Class II bacteriocins are further subdivided into pediocin-like bacteriocins (class IIa), two-peptide bacteriocins (class IIb), circular bacteriocins (class IIc) and those that cannot be assigned to any of the other bacteriocin subgroups (class IId)^2, 8^. Class IIa bacteriocins are the most extensively studied and are characterized by their strong inhibitory effect on *Listeria* sp.^9^, with pediocin PA-1 being the best-known of this class.

Generally, the production of class II bacteriocins requires several genes clustered into one or more operons encoded either on plasmids or on the chromosome. A typical gene cluster consists of a structural gene that encodes the prepeptide (or two structural genes for the two-peptide bacteriocins), and individual genes that encode an immunity protein, an ATP-binding cassette (ABC) transporter, an accessory protein for extracellular translocation of the bacteriocin, and in several cases, two regulatory proteins^10^. Bacteriocins are usually ribosomally synthesized as inactive prepeptides with an N-terminal leader sequence which is attached to a C-terminal propeptide. The leader peptide is often cleaved at a double-glycine proteolytic processing site during export by an ABC transporter and the mature peptide is released into the external environment. However, a few bacteriocins contain an N-terminal *sec*-dependent sequence leader and are secreted by general sec-dependent export systems^11–13^.

Characterization of bacteriocins and assessment of their applications often requires that they are obtained in a pure form from the culture medium or by chemical synthetic production. However, bacteriocin yields from the native host are often very low and the methods involved in their purification and chemical synthesis can be laborious and expensive^14–16^. The development of heterologous expression systems for bacteriocin production in different hosts such as *E*. *coli*, LAB and yeasts offers several advantages over native systems, including increased production levels, control of bacteriocin gene expression and characterization of bacteriocin systems from genera or species where there are no genetic tools available^17–19^. Compared to other hosts, *E*. *coli* is attractive because of its extensive genetic characterization and the availability of versatile cloning tools and expression systems. Indeed, there are many examples of the cloning of genes involved in the biosynthesis of class II bacteriocins in *E*. *coli*, such as mesentericin Y105^20^, gassericin A^21^, piscicolin 126^22^, pediocin PA-1 and AcH^23–27^, divercin V41^17, 27, 28^, enterocin A^29^, enterocin P^30^, enterocin CRL35^31^ and sakacin P^16^. However, there can also be some limitations to using *E*. *coli* as an expression host for LAB bacteriocins due to its different genetic background and its limited ability to facilitate extensive disulfide bond formation^17, 32^. To avoid the differences in codon usage that can impede translation due to the demand for tRNAs that are rare or lacking in the expression host, many bacteriocins have been expressed as optimized sequences, replacing the rare codons with synonymous codons, fused to a secretion signal sequence to deliver them to the cytoplasm or expressed in *E*. *coli* Rosetta^TM^ strains which supply tRNAs for the six codons rarely used in *E*. *coli* ^31^. Bacteriocins have also been expressed as thioredoxin fusion proteins in *trxB*/*gor* mutant (a thioredoxin reductase and gluthatione reductase knockout) *E*. *coli* to enhance solubility and the establishment of disulfide bonds^17, 22^. However, using bacteriocin fusion proteins requires an additional cleavage step and further purification to access the mature bacteriocin.

Here, we report the heterologous expression of two disulphide bond-containing class II bacteriocins, the recently described class IId bactofencin A^8^ and the well-known class IIa pediocin PA- 1^33^, directly into the medium, by cloning their native genes from the natural producers without additional *trx* gen or signal secretion sequences. This represents the first report of bactofencin A production in a Gram-negative organism. In addition, we identify the minimum gene cluster required for the expression of the respective bacteriocins in *E*. *coli* and open up new perspectives for the biotechnological production and modification of these and other bacteriocins.

## Results

### Cloning of bactofencin A (*bfn*) and pediocin PA-1 (*ped*) gene clusters in *E*. *coli*

**Figure 1:**
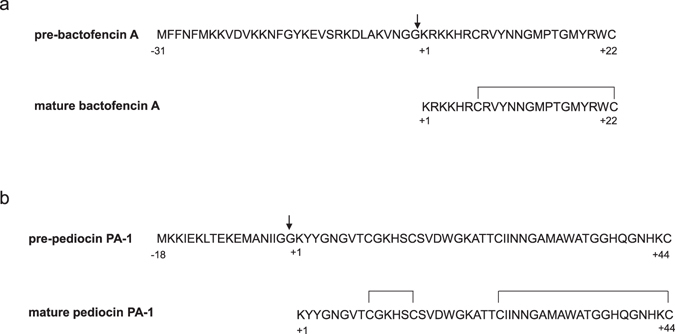
Amino acid sequence of (**a**) pre-bactofencin A and (**b**) pre-pediocin PA-1 and the mature sequences formed after processing of the leader peptide and oxidation of the cysteines. The black arrow indicates the leader peptide cleavage site.

Bactofencin A, produced by *Lactobacillus salivarius* DPC6502, is a highly basic 22-amino-acid peptide containing an intramolecular disulfide bond between Cys7 and Cys22 (Fig. 1a), with activity against pathogenic species including *Staphylococcus aureus* and *Listeria monocytogenes* ^8^. Pediocin PA-1 is a 44 amino acid peptide with a strong anti-*Listeria* activity containing four cysteine residues which form two disulfide bonds between Cys9 and Cys14 and between Cys24 and Cys44 (Fig. 1b)^33^ produced by several strains of *Pediococcus* and *Lactobacillus* species^15^.

Initial attempts to express bactofencin A in *E*. *coli* under a constitutive promoter were unsuccessful in that growth of the bacterium was retarded and the plasmid was rapidly lost. To circumvent this problem, the bactofencin A gene cluster (4,110 bp) from *Lb*. *salivarius* DPC6502 and the pediocin PA-1 (3,386 bp) operon from *Pediococcus acidilactici* LMG 2351 and different combinations of the genes within these operons were cloned into pMPB1 (Figs S1 and S2), a modified pETcoco^TM^-2 (Novagen) vector. The plasmid pETcoco^TM^-2 has been designed for cloning and expression of toxic proteins through dual control of expression: at transcriptional level by the T7 phage promoter inducible by IPTG, and for amplification at the DNA replication level by L-arabinose^34^. To generate the bactofencin A- and pediocin PA-1-operon containing plasmids, the genes of interest were amplified by PCR, assembled and joined with restriction enzyme-digested pMPB1 vector by an In-Fusion reaction. Through this process the entire bactofencin A (pMPB1-*bfnAbfnIDLSL_0052DLSL_0053*) and pediocin PA-1 (pMPB1-*pedApedBpedCpedD*) gene clusters were successfully cloned under the control of T7*lac* promoter. In order to evaluate the effect of the expression of one or more genes on heterologous bacteriocin production, 16 chimeric plasmids containing different combinations of genes were constructed (Table 1, Fig. S2).

**Table 1:** Bacterial strains and plasmids used in this work.

Strain or plasmid	Characteristics	Reference/source
**Strains**		
*Listeria innocua* DPC3572	Indicator organism	52
*Lactobacillus salivarius* DPC6502	Bactofencin A producer	52
*Pediococcus acidilactici* LMG2351	Pediocin PA-1 producer	53
*Lactobacillus delbrueckii* subsp. *bulgaricus* LMG6901	Indicator organism	LMG
*Escherichia coli* HST08 Stellar^TM^ strain	F−, *endA1*, *supE44*, *thi-1*, *recA1*, *relA1*, *gyrA96*, *phoA*, *Φ80d lacZΔ M15*, *Δ* (*lacZYA* - *argF*) *U169*, Δ (*mrr* - *hsdRMS* - *mcrBC*), *ΔmcrA*, λ−	Takara Bio USA
*Escherichia coli* BL21 Tuner^TM^ (DE3) strain	F^−^ *ompT hsdS*_B_ (r_B_^−^ m_B_^−^) *gal dcm lacY1* (DE3)	Novagen
**Plasmids**		
pETcoco^TM^-2	Plasmid with dual controls for amplification (araC-P_*BAD*_ promoter) and expression (T7*lac* promoter). Ap^r^.	Novagen^34^
APC2313	pMPB1, pETcoco^TM^-2 with a modified MCS. Ap^r^.	This work
APC2315	pMPB1- *bfnAbfnIDLSL_0052DLSL_0053*. Ap^r^.	This work
APC2316	pMPB1-*bfnA*. Ap^r^.	This work
APC2317	pMPB1- *bfnAbfnI*. Ap^r^.	This work
APC2318	pMPB1- *bfnAbfnIDLSL_0052*. Ap^r^.	This work
APC2319	pMPB1- *bfnIDLSL_0052DLSL_0053*. Ap^r^.	This work
APC2320	pMPB1- *bfnAbfnIDLSL_0053*. Ap^r^.	This work
APC2321	pMPB1- *bfnADLSL_0052DLSL_0053*. Ap^r^.	This work
APC2322	pMPB1- *bfnADLSL_0052*. Ap^r^.	This work
APC2323	pMPB1- *bfnADLSL_0053*. Ap^r^.	This work
APC2658	pMPB1- *pedApedBpedCpedD*. Ap^r^.	This work
APC2659	pMPB1-*pedA*. Ap^r^.	This work
APC2660	pMPB1- *pedApedB*. Ap^r^.	This work
APC2661	pMPB1- *pedApedBpedC*. Ap^r^.	This work
APC2662	pMPB1- *pedBpedCpedD*. Ap^r^	This work
APC2663	pMPB1- *pedApedBpedD*. Ap^r^	This work
APC2664	pMPB1- *pedApedCpedD*. Ap^r^	This work
APC2665	pMPB1-*pedApedC*. Ap^r^	This work
APC2666	pMPB1- *pedApedD*. Ap^r^	This work

^*^MCS, Multiple Cloning site. Ap^r^, ampicillin resistant, LMG, Laboratorium voor Microbiologie Culture Collection, Ghent, Belgium.

### Induction conditions for bactofencin A and pediocin PA-1 expression in *E*. *coli*

To determine the optimum time required for bacteriocin production using the T7*lac* promoter system, *E*. *coli* Tuner^TM^ (DE3) cells carrying the control plasmid (pMPB1) and *bfn* vector (pMPB1-*bfnAbfnIDLSL_0052DLSL_0053*) were grown in the presence of glucose (low-copy state (LC)) or arabinose (medium-copy state (MC)). Since IPTG concentrations above 100 µM compromised the growth of the producer strains after 24 hours (data not shown), the time for optimum production was determined by collecting samples each hour for 8 hours and at 24 hours in the presence of 25 µM of IPTG. The results showed bactofencin A activity between 2 and 8 hours after induction in LC conditions and between 1 and 4 hours in the MC state (Fig. S3). Therefore, subsequent experiments were performed following 3 hours of induction to ensure bacteriocin expression.

**Figure 2:**
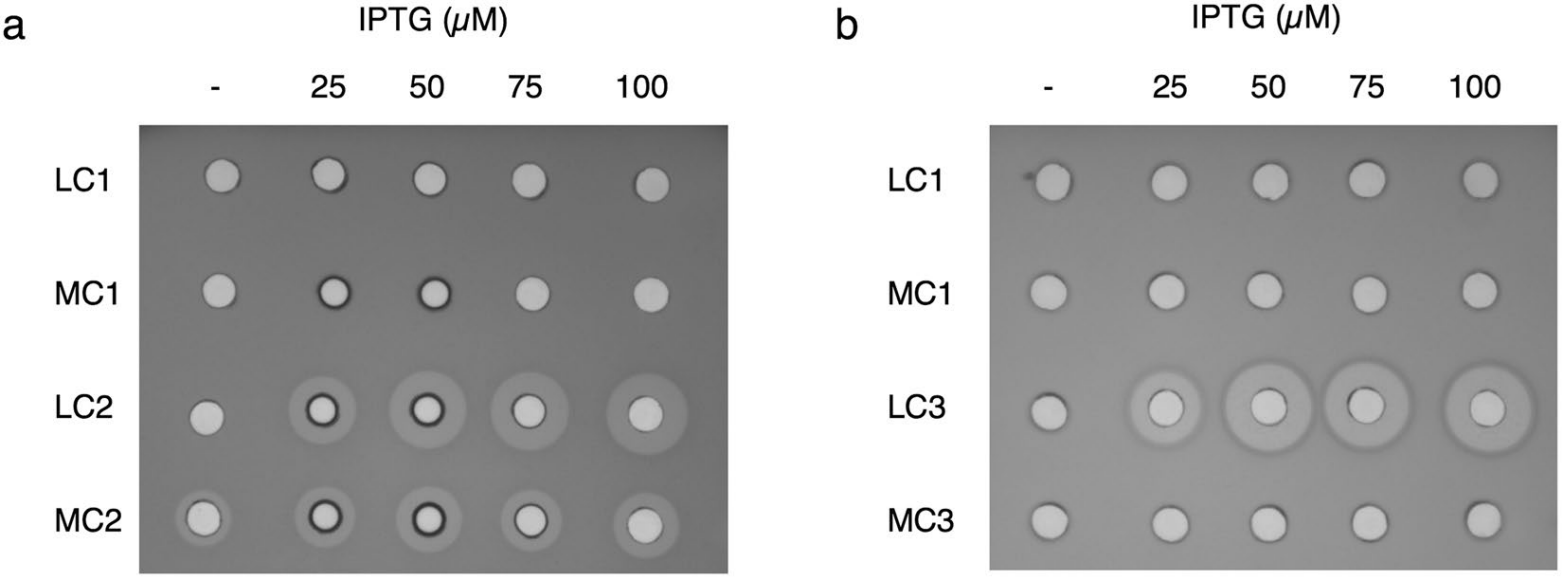
Production of bactofencin A and pediocin PA-1 in *E*. *coli*. Antibacterial activity of (**a**) bactofencin A and (**b**) pediocin PA-1 produced by supernatants of *E*. *coli* Tuner^TM^ (DE3) carrying the control (LC1, MC1), *bfn* (LC2, MC2) and *ped* (LC3, MC3) vectors at different levels of plasmid amplification (low copy (LC) and medium copy (MC)) with 0, 25, 50, 75 and 100 µM of IPTG after 3 h of induction. *Lb*. *bulgaricus* LMG6901and *L*. *innocua* DPC3572 were used as the indicator strains for bactofencin A and pediocin PA-1 activity, respectively.

To control both plasmid copy number and expression, *E*. *coli* Tuner^TM^ (DE3) cells transformed with the control plasmid, *bfn* and *ped* (pMPB1-*pedApedBpedCpedD*) vectors, were grown in LB supplemented with glucose or arabinose for 3 hours in the presence of 0, 25, 50, 75 and 100 µM of IPTG. The results demonstrate that the expressed bactofencin A in the cell-free extracellular fraction is biologically active against the indicator *Lactobacillus delbrueckii* subsp. *bulgaricus* LMG6901 in both low copy (LC2) and medium copy (MC2) states under the IPTG assay concentrations (Fig. 2a). The MC2 condition also showed an inhibitory zone when the system was not induced, suggesting a basal bactofencin A expression level. However, the activity of supernatants in the presence of IPTG was lower than those expressed at LC at the same IPTG concentration. As shown in Fig. 2b, the production of active pediocin PA-1 against *Listeria innocua* DPC3572 was only achieved at low copy level (LC3), increasing from 25 µM to 50 µM IPTG concentration.

Taken together, these results demonstrate the production of active bactofencin A and pediocin PA-1 directly into the extracellular medium by *E*. *coli* Tuner^TM^ (DE3), with the low copy state being the most suitable condition for the expression of both bacteriocins. In addition, 25 µM IPTG was selected for the induction of bacteriocin production as it was the lowest concentration that showed activity.

### Expression of the bacteriocin transporter genes is essential for *bfn* and *ped* activity in *E*. *coli*

*Bfn* and *ped* operons are composed of four genes that encode the structural peptide (*bfnA*, *pedA*), the immunity protein (*bfnI*, *pedB*), an ABC transporter (*DLSL_0052*, *pedD*) and an accessory protein (*DLSL_0053*, *pedC*). To determine the genes required for heterologous bacteriocin production, two different groups of plasmids were constructed, vectors from which had each gene individually deleted and vectors designed to express the structural gene in the presence of the immunity, transporter or accessory encoded genes.

**Figure 3:**
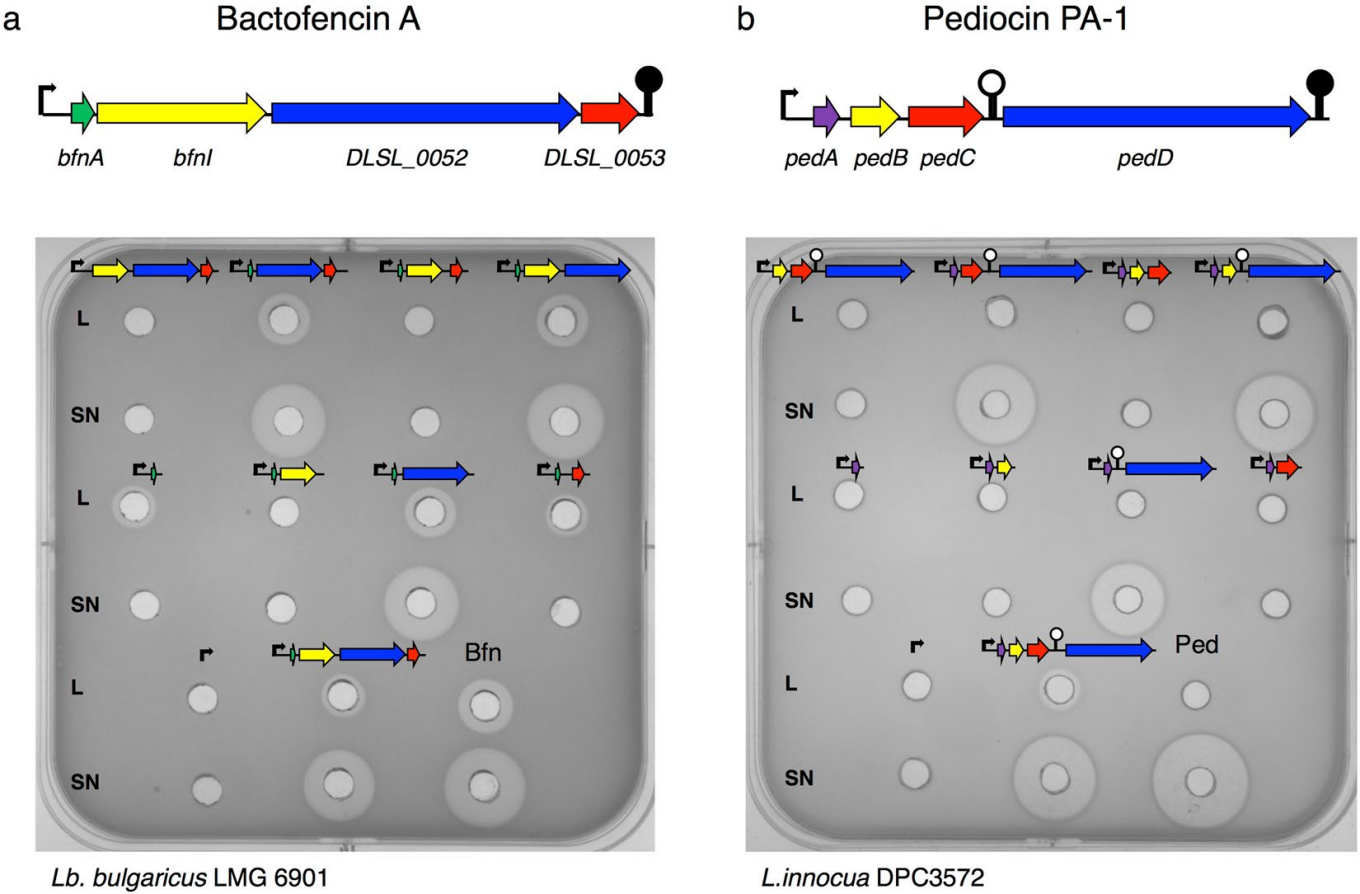
Antimicrobial activity of cell-free supernatants (SN) and lysates (L) of *E*. *coli* Tuner^TM^ (DE3) after 3 hours of 25 µM IPTG induction. (**a**) Analysis of bactofencin A activity against *Lb*. *bulgaricus* and (**b**) pediocin PA-1 activity againt *L*. *innocua*. The genes that encode the structural peptide of bactofencin and pediocin are indicated in green and purple arrows, respectively. The immunity, the transporter and the accessory protein encoded genes are shown in yellow, blue and red arrows. The black arrow indicates the T7*lac* promoter. Samples from the natural producers *Lb*. *salivarius* DPC6502 and *P*. *acidilactici* LMG2351 are indicated as Bfn and Ped.

The results revealed that the cell-free supernatants contained active antimicrobials exclusively in the presence of the transporters but independent of the presence of the accessory gene in both cases (Fig. 3). Interestingly, small zones of inhibition were also observed in the lysates of *bfn* constructions containing the structural gene alone or in combination with the *DLSL_0053* gene (Fig. 3a). In the case of pediocin PA-1, only the lysate of the strain containing the whole *ped* operon showed activity (Fig. 3b).

**Figure 4:**
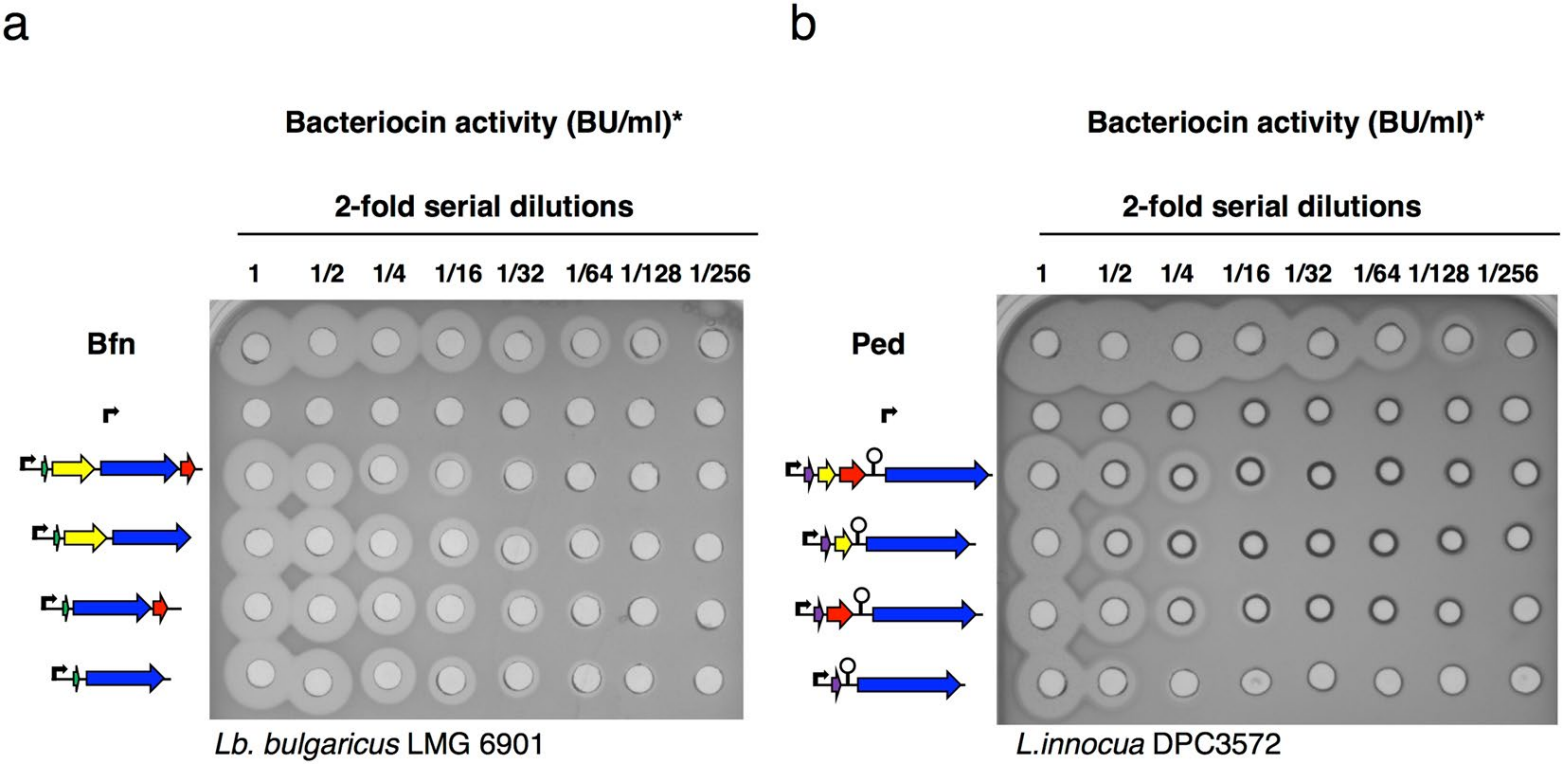
Relative quantification of cell-free supernatants (Bacteriocin units (BU)/ml). Antimicrobial activity of (**a**) bactofencin A and (**b**) pediocin PA-1 produced by *E*. *coli* transformed with the plasmids APC2313 (control), APC2315, APC2318, APC2321 and APC2322, and APC2658, APC2663, APC2664 and APC2666, respectively, in comparison with the samples from the natural producers *Lb*. *salivarius* DPC6502 (Bfn) and *P*. *acidilactici* LMG2351 (Ped). *Bacteriocin activity (BU/ml) was calculated as the inverse of the highest dilution showing inhibition of indicator strains divided by volume (0.05 ml).

The quantification of the relative extracellular bactofencin A activity produced by *bfn* constructs was 2-fold higher (640 BU/ml) than that produced by the whole *bfn* operon under the conditions assayed (Fig. 4a). By contrast, the pediocin PA-1 constructions showed the same production level in the whole operon vector and *pedB* mutant (160 BU/ml) and a 2-fold reduction in *pedC* and *pedCD* mutants (Fig. 4b), indicating that *pedC* is probably involved in the optimal activation of pediocin PA-1 produced by *E*. *coli*. Although the amount of bactofencin A and pediocin PA-1 produced by *E*. *coli* was found to be 4 and 8-fold lower than the bacteriocin produced by an overnight culture of the natural producers, it should be noted that this level of bacteriocin production in *E*. *coli* is reached in only 3 hours. In addition, these results suggest that the bactofencin A operon can be simplified for heterologous expression, requiring only the transporter gene and the structural gene itself.

### Purification of the active bfn and ped supernatants from *E*. *coli* and MALDI TOF mass spectrometry analysis of bacteriocin

**Figure 5:**
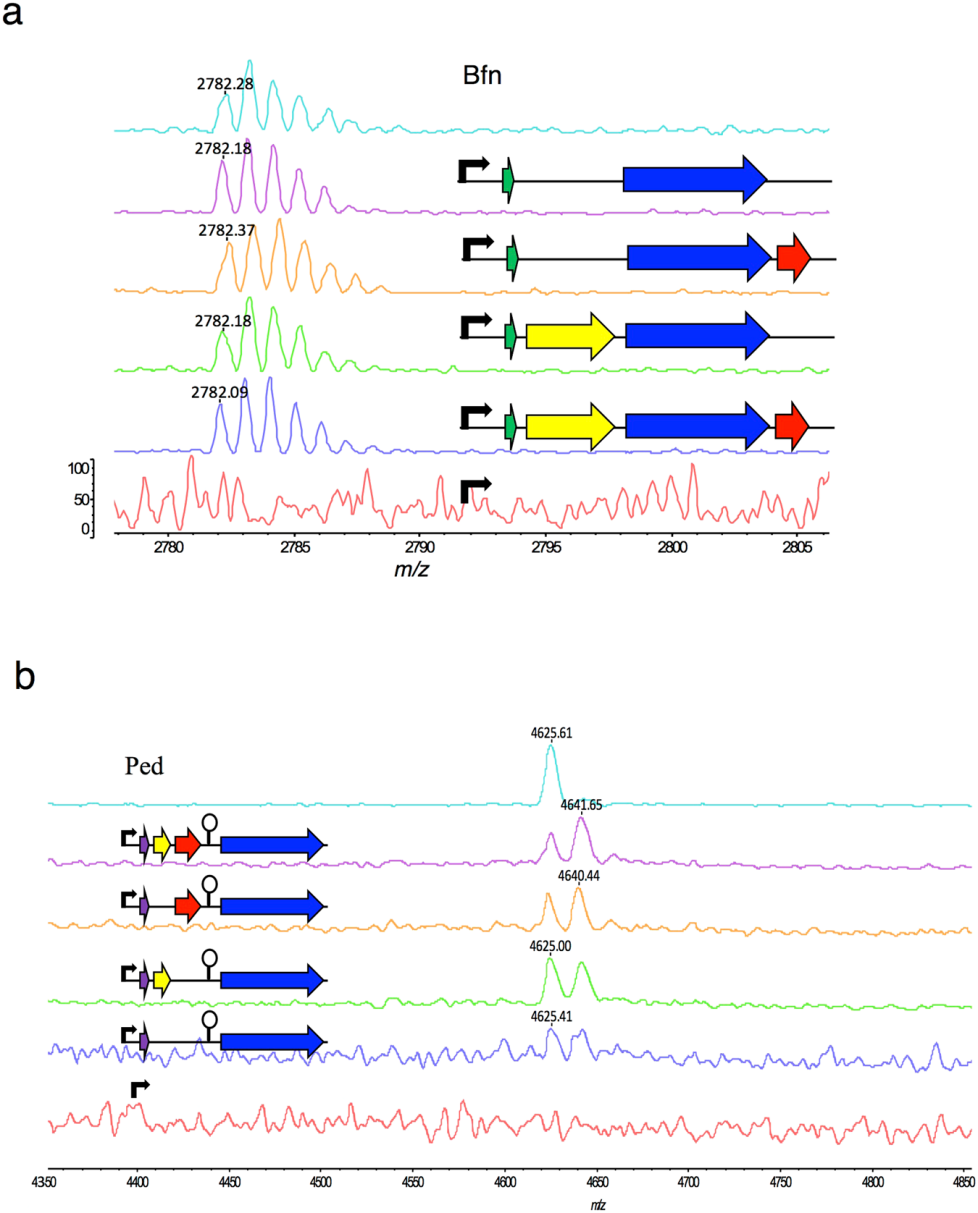
MALDI-TOF mass spectrometry analysis. (**a**) Soluble extracellular fractions from bactofencin A *E*. *coli* producers and (**b**) pediocin PA-1 producers after 3 hours of IPTG induction. Samples from the natural producers *Lb*. *salivarius* DPC6502 and *P*. *acidilactici* LMG2351 are indicated as Bfn and Ped.

The sequence analysis of *DLSL_0053* (bactofencin A transport accessory gene) revealed the presence of the cd02947, a thioredoxin family domain, suggesting that this protein is involved in disulfide bond formation. To evaluate the presence of a disulfide bond in the active *bfn*, the supernatants from the natural producer *Lb*. *salivarius* and Tuner^TM^(DE3) *E*. *coli* carrying the control plasmid, the entire *bfn* operon, and the constructs without the accessory gene (pMPB1- *bfnAbfnIDLSL_0052*), the immunity gene (pMPB1*- bfnADLSL_0052DLSL_0053*) and without both (*pMPB1- bfnADLSL_0052*) were purified and analysed for the presence of bactofencin A by matrix-assisted laser desorption ionization-time of flight mass spectrometry (MALDI-TOF MS) in positive ion reflectron mode. Interestingly, the results showed that all fractions, with the exception of the negative control, contained the 2,782 Da bactofencin A mass, indicating that the intramolecular disulfide bond formed in all cases, independent of the presence of the accessory gene (Fig. 5a).

MALDI TOF analysis in reflectron mode was also used to confirm the presence of both disulphide bonds in pediocin PA-1 constructs but the concentration of sample pMPB1-*pedApedD* was too low to successfully detect the monoistopic mass of pediocin in this sample. Instead pediocin PA-1 samples were analysed in positive ion linear mode where ions were averaged. MALDI TOF MS analysis of pediocin PA-1 fractions from *E*. *coli* showed that in addition to the expected mass of 4,625 Da, corresponding to the pediocin PA-1 synthesized by the natural producer *P*. *acidilactici*, a 4641 Da mass, consistent with the oxidation of methionine, was also detected (Fig. 5b).

### Increasing the heterologous production of *bfn* in *E*. *coli*

**Figure 6:**
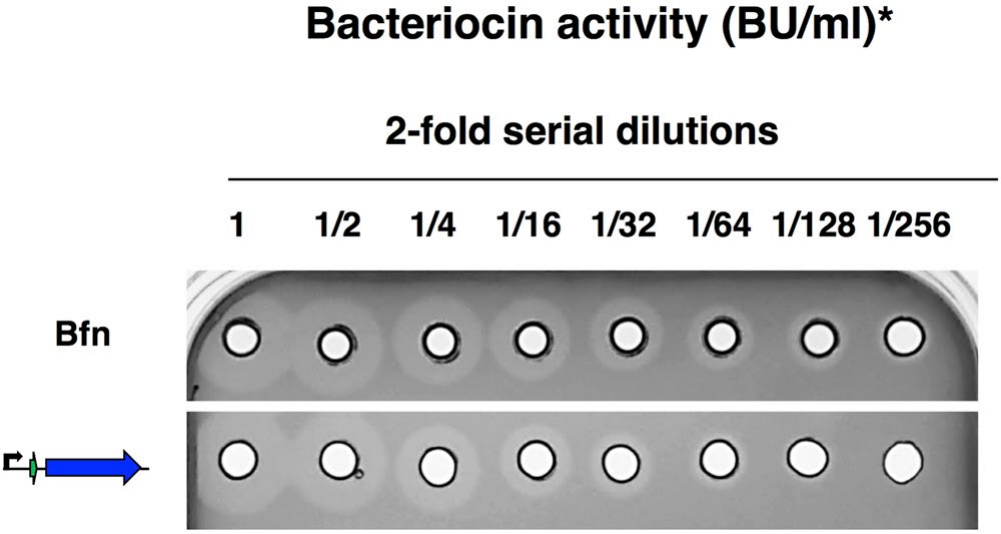
Quantification of the expression of bactofencin A produced by *E*. *coli* Tuner (DE3) transformed with the plasmid containing the structural and the transporter encoded genes after 3 h of 100 µM ITPG induction. The activity of the supernatant from an overnight culture of *Lb*. *salivarius* is indicated as Bfn. *Bacteriocin activity (BU/ml) was calculated as the inverse of the highest dilution showing inhibition of *Lb*. *bulgaricus* indicator strain divided by volume (0.05 ml).

The level of production of active bactofencin A using the simplest system, consisting of both structural and transporter genes, was 640 BU/ml under 25 µM of IPTG of induction. This corresponds to approximately 25% of the amount synthesized by the natural producer *Lb*. *salivarius* DPC6502 and prompted investigations to determine if the synthesis of active bacteriocin could be improved by increasing the inducer concentration. To do that, *E*. *coli* Tuner^TM^ (DE3) transformed with pMPB1-*bfnADLSL_0052* culture, was induced with 100 µM IPTG and the activity of the supernatant analysed after 3 h of induction. As shown in Fig. 6, the relative quantity of active bactofencin A in 3 hours reached approximately 50% of that produced by the natural producer in an overnight culture.

## Discussion

Bacteriocins produced by LAB offer a great variety of different applications for the food, health and pharmaceutical industries^5, 35^. However, the high cost of bacteriocin purification from natural producers, or chemical synthesis, can limit their large-scale production. This has led to the exploration of alternative heterologous expression hosts such as *E*. *coli*. To achieve high-level production of native bacteriocins in this host it is also necessary to express the specialized secretion machinery required for the recognition and processing of their prepeptides. However, the use of systems involving, for example, T7 RNA polymerase can result in cellular toxicity arising from overexpression of secretion machinery and other integral membrane proteins^26^. For these reasons, in this study pETcoco^TM^-2 was selected as a backbone vector to overexpress the bactofencin A and pediocin PA-1 operons. The dual replicon nature of this vector facilitates cloning under the T7*lac* promoter and expression of toxic proteins due to the extremely low basal expression level in the single copy state which promotes the stability of recombinant plasmids, while allowing the amplification of plasmid DNA copy number by arabinose induction and/or the increase of protein expression by adding IPTG. In this work, the PCR-amplified genes of interest from *Lb*. *salivarius* DPC6502 and *P*. *acidilactici* LMG2351, encoding bactofencin A and pediocin PA-1 operons, respectively, were cloned into the linearized pMPB1 vector, based on the commercial plasmid pETcoco^TM^-2. This facilitated the successful generation of stable expression vectors with the entire *bfn* and *ped* operons as well as the gene-deleted mutants by assembling the remaining genes of interest, avoiding laborious gene inactivation approaches. In addition, the use of pETcoco^TM^-2 derived vectors has allowed the identification of the optimal conditions (low copy number and <100 μM IPTG concentration) for each bacteriocin expression without compromising bacterial growth. Interestingly, the production of active bacteriocin in the transformed *E*. *coli* Tuner^TM^ (DE3) cells was not proportional to copy number, implying that the bactofencin A antimicrobial activity decreased at the medium-copy state and it became undetectable in the pediocin PA-1 constructs. Considering that the growth rate of the cultures was similar to the control (data not shown), the induction of plasmid copy number seems to interfere with the ability of *E*. *coli* to overproduce these bacteriocins. This can be explained by many factors, including the structural features of the recombinant gene sequences, the stability and translational efficiency of the mRNA, incomplete processing of signal sequences, the metabolic burden on the host strain, the capacity of the transport system and the potential toxicity of the bacteriocin^14, 36–38^. Thus, a low copy vector was the most suitable condition for evaluating the expression of both bacteriocins.

Previous genome sequencing of *Lb*. *salivarius* DPC 6502 suggested that bactofencin A biosynthesis involves four genes *bfnA*, *bfnI*, *DLSL_0052* and *DLSL_0053* organized in a single operon^8^. In the present work, we obtained a full reconstitution of the *in vivo* synthesis of bactofencin A in a Gram-negative host. Tuner^TM^ (DE3) *E*. *coli* cells harbouring the complete *bfn* gene cluster successfully inhibited *Lb*. *bulgaricus*. Moreover, the analysis of expression of gene-deleted mutants demonstrated that only the putative ABC transporter (DLSL_0052) is essential for bacteriocin export into the extracellular medium. The ABC transporter contains an N-terminal peptidase C39 domain responsible for the cleavage of double-glycine leader sequences^10, 39^. In the absence of the transporter, it was not possible to detect antimicrobial activity in supernatants, supporting the theory that this protein plays an essential role in the processing and secretion of the mature active bacteriocin. In addition, some activity was found in the lysate of *E*. *coli* carrying plasmids, encoding the single structural peptide together with the putative bactofencin A transport accessory protein (DLSL_0053), suggesting that a small amount of prepeptide could be processed by proteases present in the host. The sequence analysis of DLSL_0053 revealed the presence of cd02947, a thioredoxin family domain involved in the formation of disulfide bonds. However, the *DLSL_0053* gene deletion proved that this protein is not required for bactofencin A activity in *E*. *coli*. The molecular mass of bactofencin A from active supernatants detected by MALDI-TOF MS analysis was 2,782 Da in all the vectors including those without the transporter accessory gene, indicating the presence of a disulfide bond. Considering this result, it could be hypothesized that the processed peptides could go through the periplasm which contains the enzyme DsbA which catalyzes disulfide bond formation^40^.

The present study shows that the production of pediocin PA-1 also requires the presence of the structural gene and the ABC transporter. This is in contrast to the results obtained by Venema *et al*.^41^ who concluded that PedC was also essential for pediocin PA-1 secretion, and supports the results reported by other authors^24, 25^. However, it is of interest to note that the amount of active pediocin produced by *E*. *coli* increases in the presence of *pedC*, suggesting that the accessory protein PedC enhances active pediocin PA-1 production in *E*. *coli*. These results are in accordance with those described previously by Oppegard *et al*.^42^ who indicated that PedC is important to ensure the correct formation of disulfide bonds in pediocin PA-1. In contrast, MALDI-TOF MS analysis of the purified pediocin PA-1 supernatants from *E*. *coli* showed an additional peptide fragment of 4,641 Da that is not present in the fractions from the natural producer *P*. *acidilactici* (4,625 Da). The 16 Da molecular mass difference is explained by the oxidation of methionine residues to MetSO during the recombinant production as it has been described in other heterologous hosts such as yeasts^43, 44^ and LABs^45–47^. To protect the peptide from oxidation, Met could be replaced by either Ala, Ile or Leu, with minor effects on the bacteriocin activity^48^.

In general, overexpressed recombinant proteins in *E*. *coli* are accumulated either in the periplasmic space or in the cytoplasm in inclusion bodies so additional purification steps are required to obtain pure active peptide^49^. In contrast, we show that the use of derived pETcoco-2 vectors together with the Tuner^TM^ (DE3) *E*. *coli* host allows the expression of active bacteriocins directly into the medium and, therefore, could be used to simplify the purification process. Remarkably, both secreted bacteriocins contained disulfide bonds, overcoming one of the major limitations of disulfide bond production in *E*. *coli*.

This is, to our knowledge, the first description of the production of bactofencin A in *E*. *coli* and the functional analysis of the genes involved in their heterologous production. The analysis revealed that only the structural gene and the transporter encoded gene are required for expression of fully functional bacteriocins. Induction with 100 µM IPTG led to an increase in the bactofencin A production, reaching in 3 hours 50% of the bactofencin A produced by the natural producer *Lb*. *salivarius* in an overnight culture. Even though the levels of heterologous production of bactofencin A and pediocin PA-1 in *E*. *coli* were lower than those observed in the natural producer, its recovery from the supernatants may be faster, easier and more cost effective.

The results presented here suggest that this system could be useful for expressing new bacteriocin operons described *in silico* as well as their characterization at molecular level when there is no access to the original producer strains or the transformation efficiencies of natural producers is a severe impediment for gene analysis, opening the door to the heterologous expression and harnessing of the potential of new bacteriocins in the future.

## Methods

### Bacterial strains, plasmids and culture conditions

Bacterial strains and plasmids used in this study are listed in Table 1. *Escherichia coli* HST08 strain Stellar^TM^ (Takara Bio USA, Inc, Mountain View, CA), was used for standard cloning procedures and *Escherichia coli* BL21 strain Tuner^TM^ (DE3) (also called *E*. *coli* Tuner^TM^ (DE3)) (Novagen, EMD Millipore, Billerica, MA) was used for gene expression experiments. *Escherichia coli* strains were grown aerobically at 180 r.p.m. and 37 °C in Luria-Bertani (LB) medium and supplemented with ampicillin (50 μg/ml) for the selection and maintenance of plasmids. *Lactobacillus salivarius* DPC6502, used as a bactofencin A producer, and the bactofencin A-sensitive strain *Lactobacillus delbrueckii* subsp. *bulgaricus* LMG6901 (*Lb. bulgaricus* LMG 6901) were grown under anaerobic conditions at 37 °C in MRS medium (Difco Laboratories, Detroit, MI). Anaerobic conditions were maintained with the use of anaerobic jars and Anaerocult A gas packs (Merck, Darmstadt, Germany). *Pediococcus acidilactici* LMG2351, used as a pediocin PA-1 producer, was cultured aerobically in MRS broth at 30 °C without shaking. The pediocin PA-1-sensitive indicator strain *Listeria innocua* DPC 3572 was grown aerobically at 37 °C in brain heart infusion (BHI) medium (Merck) without shaking. Gene expression was induced by IPTG (isopropyl- β-D-thiogalactopyranoside). IPTG was supplied by Fisher Scientific (Dublin, Ireland) dissolved in water, filter sterilized, and added to the media. (D)  + -glucose, L- + -Arabinose and other chemical reagents were obtained from Sigma Aldrich (Arklow, Ireland). *Pediococcus acidilactici* LMG2351 was a gift from Dzung Bao Diep, Laboratory of Microbial Gene Technology, Agricultural University of Norway, Ås, Norway.

### Molecular biology general procedures

All DNA manipulations were performed following standard protocols^50^. All oligonucleotides used in this study are described in Supplementary Tables S1 and S2. The plasmid pETcoco^TM^-2 (Novagen) was used for gene construction and expression. Firstly, the multiple cloning site (MCS) was replaced by the product of annealing of MCS F SphI and MCS R AvrII primers, digested with SphI-AvrII and cloning into pETcoco^TM^-2 SphI-AvrII, generating plasmid pMPB1 (APC2313). A detailed description of the cloning of the bactofencin A and pediocin PA-1 gene fragments into the linearized pMPB1 is provided in the Supplementary information. The primers were designed according to the instructions of In-Fusion HD cloning and they were obtained from Sigma Aldrich (Arklow, Ireland). Briefly, the bactofencin A and pediocin PA-1 sequences were amplified by polymerase chain reaction (PCR) using CloneAmp HiFi PCR premix (Takara Bio USA, Inc, Mountain View, CA) and genomic DNA from *L*. *salivarius* DPC 6502 and plasmid DNA from *P*. *acidilactici* LMG2351 were used as DNA templates, respectively. The PCR products were eluted with illustra GFX PCR DNA and Gel Band Purification kit (GE Healthcare, Buckinghamshire, UK) and inserted into the linearized pMPB1 vector using In-Fusion HD cloning Plus (638910, Takara Bio USA, Inc, Mountain View, CA) as described previously^51^. The constructions were transformed into Stellar^TM^ competent cells and colonies were selected on plates containing ampicillin 50 μg/ml) and D (+)-glucose (0.2%). The transformants were confirmed by colony PCR reactions. Then, the number of copies of the recombinant plasmids was increased by L-arabinose induction before plasmid DNA isolation was analysed by double digestion and sequencing. All DNA sequencing were performed by GATC Biotech (Koln, Germany). The resulting vectors are summarised in the Table 1 and Fig. S2. Restriction enzymes and T4 DNA ligase were obtained from New England Biolabs (Ipswich, M). Genomic and plasmid DNA were isolated using GenElute^TM^ Bacterial Genomic DNA kit (Sigma Aldrich, St.Louis, MO) and NucleoSpin® plasmid kit (Macherey-Nagel, Duren, Germany), respectively.

### Induction of bacteriocin expression

Bactofencin A and pediocin PA-1 production were tested under different expression conditions (plasmid copy number and IPTG concentration) in order to find the best condition to induce bacteriocin production. For this purpose, Tuner^TM^ (DE3) *E*. *coli* cells were transformed with the control vector (pMPB1) and those carrying *bfn* (pMPB1- *bfnAbfnIDLSL_0052 DLSL_0053*) and *ped* operons (pMPB1-*pedApedBpedCpedD*), and isolated colonies were grown in LB broth containing ampicillin (50 μg/ml) and 0.2% D(+)-glucose (LBAp50Glc0.2), which maintains the plasmid in a single-copy state, for 16 hours at 37 °C with shaking. The cultures were diluted 1:50 into fresh LBAp50Glc0.2 medium to keep the low copy state (LC) and fresh LB containing ampicillin (50 μg/ml) and L-+-Arabinose (0.01%) to increase the number of copy of plasmids (Medium Copy state (MC)). When cells reached OD_600_ 0.5–0.6, IPTG was added at concentrations of 0, 25, 50, 75 and 100 µM and the cultures were incubated for 3 h at 37 °C before determining the antimicrobial effect of the supernatants against the indicator strains as described in bacteriocin activity assay.

### Bacteriocin activity assay

Bactofencin A and pediocin PA-1 antimicrobial activities were determined by the well-diffusion method against the indicator strain *L*. *delbrueckii* subsp. *bulgaricus* LMG6901 and *L*. *innocua* DPC3572, respectively. Briefly, 5 ml of transformed *E*. *coli* cells were centrifuged at 4,000 × *g* at 4 °C for 15 minutes. The bacterial pellets were washed and resuspended in 5 ml of PBS solution and further lysed via sonication (5 pulses at 16 W, 15 s/pulse and intervals of 45 s on ice) in a MSE Soniprep 150 (MSE, London, UK) and centrifuged at 4,000 × *g* at 4 °C for 15 min. The resulting cell-free intracellular fraction from the lysate and the cell-free supernatant of the cultures were filtered with 0.20 μm membrane filters (Sartorius, Germany). 50 μl of cell-free supernatants (SN) and lysates (L) of each culture were applied to 5 mm diameter wells on MRS 1% agar plates containing *L*. *delbrueckii* subsp. *bulgaricus* LMG6901 and BHI 0.8% agar plates containing *L*. *innocua* DPC3572. MRS and BHI agar plates were refrigerated for two hours prior to incubation at 37 °C under anaerobic and aerobic conditions, respectively, for 24 hours.

As positive controls of bactofencin A and pediocin PA-1 production, the natural producers *L*. *salivarius* DPC6502 and *P*. *acidilactici* LMG2351 were grown in MRS broth at 37 °C (under anaerobic conditions) and 30 °C (aerobic conditions) for 16 h, respectively. Cell-free culture supernatants were obtained by centrifugation of cultures at 4,000 × *g* at 4 °C for 15 min. The pH was adjusted to pH 7 using 1 M NaOH to avoid any antimicrobial activity caused by the acidity of the cell free supernatants and filtered through 0.20 μm pore-size filters. The pellets were sonicated as described previously.

To quantify the relative active bacteriocin production, 50 µl of two-fold serial dilutions of each sample were placed in wells. The bacteriocin activity was expressed in bacteriocin units per ml (BU ml^−1^) and was defined as the reciprocal of the highest dilution showing inhibition of the indicator strain.

### Purification and MALDI TOF mass spectrometry analysis of bfn and ped

Twenty five ml of culture of *L*. *salivarius* DPC 6502 and *E*. *coli* Tuner DE3 containing the bacteriocin expression plasmids were harvested by centrifugation at 4,000 × *g* at 4 °C for 20 min, the bactofencin A supernatants were applied to a column containing 2 ml SP Sepharose fast-flow cation-exchange resin (GE Healthcare, United Kingdom), equilibrated with buffer A [20 mM potassium phosphate buffer and 25% acetonitrile, pH 2.5]. The column was then washed with buffer A and with 10 ml buffer B (buffer A containing 500 mM KCl) and peptides eluted with 10 ml of Buffer C (buffer A containing 1000 mM KCl). The 1 M KCl eluents were desalted using C18 SPE zips (Millipore, Cork, Ireland). Zip tips were primed by washing with 3 × 10 µl aliquots of 100% acetonitrile followed by 5 × 10 µl aliquots of 0.1% TFA. The sample was bound to the zip tip by repeated pipetting of a 50 µl aliquot of sample, the tip was washed with 3 × 10 µl aliquots of 0.1% TFA and bactofencin A eluted by repeated pipetting in 3 µl CHCA matrix. Samples were analysed for the presence of bactofencin A by MALDI TOF mass spectrometry (Axima- TOF2; Shimadzu Biotech, Manchester, United Kingdom) in positive ion reflectron mode.

One hundred ml of pediocin PA-1 samples were applied to 500 mg 6 ml Strata C18-E SPE columns pre-equilbrated with methanol and water. The columns were washed with 6 ml 30% ethanol and the samples eluted with 6 ml of IPA (70% propan-2-ol, 0.1% TFA). The IPA eluents were analysed by MALDI TOF mass spectrometry in positive ion reflectron and linear mode.

## Electronic supplementary material

Supplementary Information
