# Supplementary material for: Controlled functional expression of the bacteriocins pediocin PA-1 and bactofencin A in Escherichia coli
Source: Sci Rep. 2017 Jun 8;7:3069. doi: 10.1038/s41598-017-02868-w (PMC5465099; doi:10.1038/s41598-017-02868-w)
Supplement: Supplementary file 1 — Supplementary Information [file 41598_2017_2868_MOESM1_ESM.doc]

**Supplementary information**

Controlled functional expression of the bacteriocins pediocin PA-1 and bactofencin A in *Escherichia coli*

**Beatriz Mesa-Pereira^1,2^, Paula M. O’Connor^1,2^,  Mary C. Rea ^1,2^, Paul D. Cotter^1,2^, Colin Hill^2,3^ & R. Paul Ross^1,2,3*^**

^1^ Teagasc Food Research Centre, Teagasc Moorepark, Fermoy, Co. Cork, Ireland

^2^ APC Microbiome Institute, University College Cork, Ireland

^3^Department of Microbiology, University College Cork, Ireland

*corresponding author E-mail: [p.ross@ucc.ie](mailto:p.ross@ucc.ie)

Address: College of Science, Engineering and Food Science (SEFS), University College Cork, Ireland

Phone: 00353214903760

**Supplementary Information**

**Methods-Construction of bactofencin A and pediocin PA-1 derivative vectors.**

pETcoco^TM^-2 (Novagen) was the starting vector whose multiple cloning site (MCS) was first modified to remove NsiI and PmlI sites and S and HSV-tags and reorganize the restriction sites. To do that, the MCS was replaced by the product of annealing of MCS F SphI and MCS R AvrII primers. Annealing was achieved with 9 µl of each 10µM oligonucleotide and 2 µl of annealing buffer (20 mM Tris HCl, pH7.4, 2 mM MgCl_2_, 50 mM NaCl). The reaction mixture was incubated at 94˚C for 10 min and gradually cooled to 25˚C. The product was digested with SphI and AvrII enzymes and ligated with pETcoco^TM^-2 digested previously with the same restriction endonucleases. The resulting plasmid was further confirmed by colony PCR, double digestion with NdeI and HindIII and sequenced using pMPB1 Fw1 and pMPB1 Rv1 primers. The vector was termed pMPB1 (APC 2313) and was used for all the constructions.

The bactofencin A operon sequence was amplified using genomic *L. salivarius* DPC6502 DNA as a DNA template. Linearized SphI pMPB1 vector was used to accept different PCR products corresponding to bactofencin A genes. The whole operon (4,145 bp) was amplified by PCR using bfn Fw1 and bfn Rv1 primers,  *bfnA* sequence (195 bp) was amplified with bfn Fw1 and pMPB1 bfnA Rv , *bfnAbfnI* (1,456 bp) with bfn Fw1 and pMPB1 bfnI Rv , *bfnAbfnIDLSL_0052* (3,734 bp) with bfn Fw1 and pMPB1 DLSL52 Rv , *bfnIDLSL_0052DLSL_0053* (3,910 bp) with pMPB1 bfnI Fw and pMPB1 DLSL53 Rv primers. They were assembled in a 2:1 (insert: vector) ratio into the SphI pMPB1 vector to construct pMPB1- *bfnAbfnIDLSL_0052DLSL_0053* (APC2315), pMPB1- *bfnA* (APC 2316), pMPB1-*bfnAbfnI* (APC 2317), pMPB1- *bfnAbfnIDLSL_0052* (APC2318), pMPB1-*bfnIDLSL_0052 DLSL_0053* (APC2319) respectively.

Two fragments of bactofencin A cluster were individually amplified by PCR with bfn Fw1 and pMPB1 bfnItail Rv (1,517 bp), and pMPB1 DLSL53 Fw2 and pMPB1 DLSL53 AvrII Rv (440 bp) primers. The fragments were joined in a 2:2:1 (insert: insert: vector) ratio with the SphI-AvrII pMPB1 to generate pMPB1-*bfnAbfnIDLSL_0053* (APC 2320). The sequences amplified with bfn Fw1 and pMPB1 bfnAtail Rv (246 bp), and pMPB1 DLSL0052 Fw2 and pMPB1 DLSL0053 AvrII Rv (2,724 bp) primers were assembled in a 10:10:1 ratio into the linearized pMPB1 to construct pMPB1-*bfnADLSL_0052DLSL_0053* (APC 2321). The 246 bp product was assembled in a 10:2:1 ratio with the fragment amplified with pMPB1 DLSL0052 Fw2 and pMPB1 DLSL0052 AvrII Rv (2,318 bp) to make pMPB1-*bfnADLSL_0052* (APC2322). The same 246 bp fragment was joined to the product of pMPB1 DLSL0053 Fw3 and pMPB1 DLSL0053 AvrII Rv primers (442 bp) in a 10:10:1 ratio to build pMPB1-*bfnADLSL_0053* (APC 2323).

Pediocin PA-1 sequence was amplified using plasmid DNA from *P. acidilactici* LMG2351as a template. The entire operon (3,379 bp) was amplified with pMPB1 pedA Fw1 and pMPB1 pedD Rv1 primers and cloned into SphI AvrII pMPB1 vector in a 5:1 ratio using In-Fusion HD Cloning enzyme to generate pMPB1-*pedApedBpedCpedD* (APC 2658). The product of amplification *pedA* (223 bp) with pMPB1 pedA Fw1 and pMPB1 pedA Rv1 primers, *pedApedB* (599 bp) with pMPB1 pedA Fw1 and pMPB1 pedB Rv1 using APC2658 plasmid DNA as a template, *pedApedBpedC* (1,147 bp) with pMPB1 pedA Fw1 and pMPB1 pedC Rv1 and *pedBpedCpedD* (3,194 bp) with pMPB1 pedB Fw1 and pMPB1 pedD Rv1 primers were assembled in a 2:1 (insert: vector) ratio into the linearized SphI-AvrII pMPB1 vector to generate pMPB1- *pedA* (APC2659), pMPB1-*pedApedB* (APC2660), pMPB1-*pedApedBpedC* (APC2661), pMPB1-*pedBpedCpedD* (APC 2662), respectively. The PCR products amplified with pMPB1 pedA Fw1 and pMPB1 pedBD Rv1 (604 bp) and pMPB1 pedBD Fw1 and pMPB1 pedD Rv1 (2,312 bp) primers were joined in a 10:5:1 ratio into SphI-AvrII pMPB1 vector to construct pMPB1-*pedApedBpedD* (APC2663). A ratio of 10:10:1 was used for the ligation of the PCR products of pMPB1 pedA Fw1 and pMPB1 pedA Rv1int (227 bp) and pMPB1 pedC Fw1int and pMPB1 pedD Rv1 (2,798 bp) primers to make pMPB1-*pedApedCpedD* (APC 2664). The same 227 bp product was joined to the product of pMPB1 pedC Fw1int and pMPB1 pedC Rv1 (565 bp) primers to generate pMPB1-*pedApedC* (APC2665). Finally, pMPB1- *pedApedD* (APC2666) was generated by the 10:10:1 ligation of the sequences amplified with pMPB1 pedA Fw1 and pMPB1 pedAD Rv1 (226 bp), and pMPB1 pedD Fw1 and pMPB1 pedD Rv1 (2,290 bp) primers.

All the transformants were analysed by colony PCR, double digestion with NcoI and NdeI and sequence with the primers included in Table S2.

**Supplementary Figure S1**

**
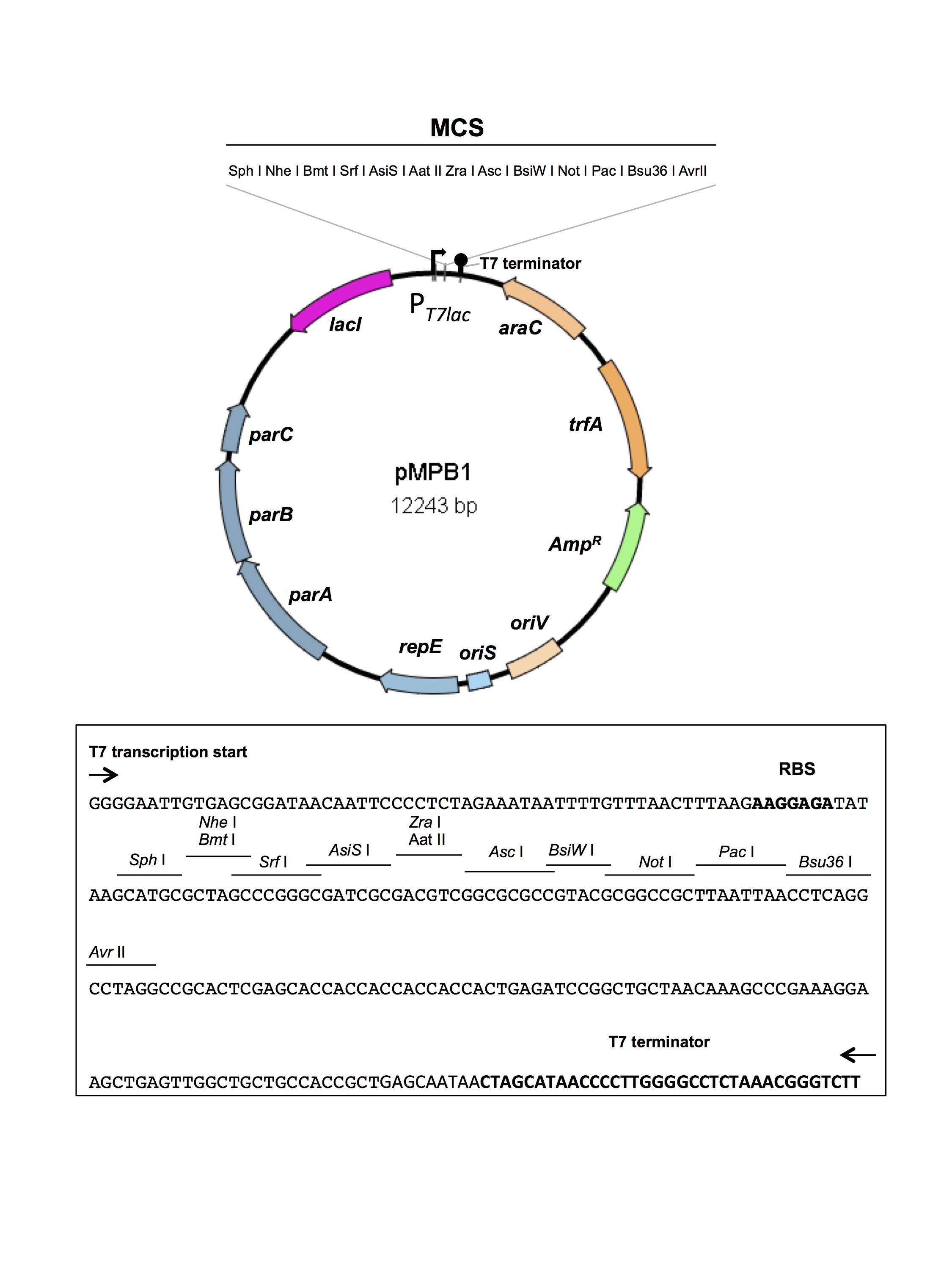
**

**Figure S1. Map of bacteriocin expression vector pMPB1**.Important features shown include the IPTG-inducible T7 RNA polymerase promoter (T7lac promoter) and associated lacI gene required for regulation. The vector is maintained in the single copy state by *oriS*, *repE* and *parABC* elements. *trfA* and *oriV* are involved in the medium copy state. Restriction sites included in the multiple cloning site (MCS) are also indicated in the sequence. RBS (Ribosome Binding Site), Amp^R^ (Ampicillin resistance gene).

/**Supplementary Figure S2**


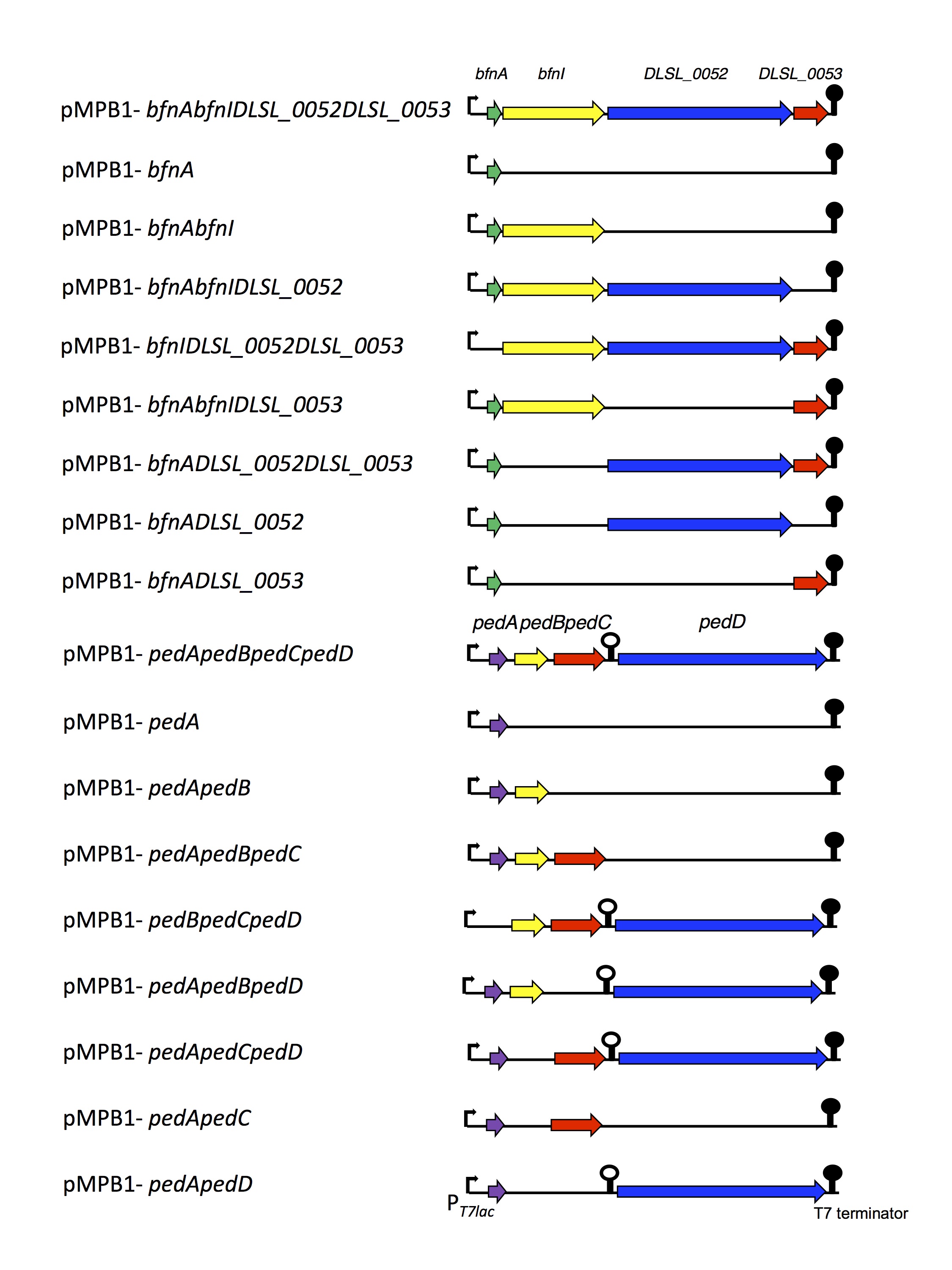


**Figure S2. Organization of gene combination contained in P*T7lac*- T7 terminator in pMPB1 derived vectors.** The genes that encode the structural peptide of bactofencin and pediocin are indicated in green and purple arrows, respectively. The immunity , the transporter and the accessory protein encoded genes are shown in yellow, blue and red arrows. Black arrow represents the T7*lac* promoter region and the black lollipop indicates the T7 transcriptional terminator.

/**Supplementary Figure S3**

**
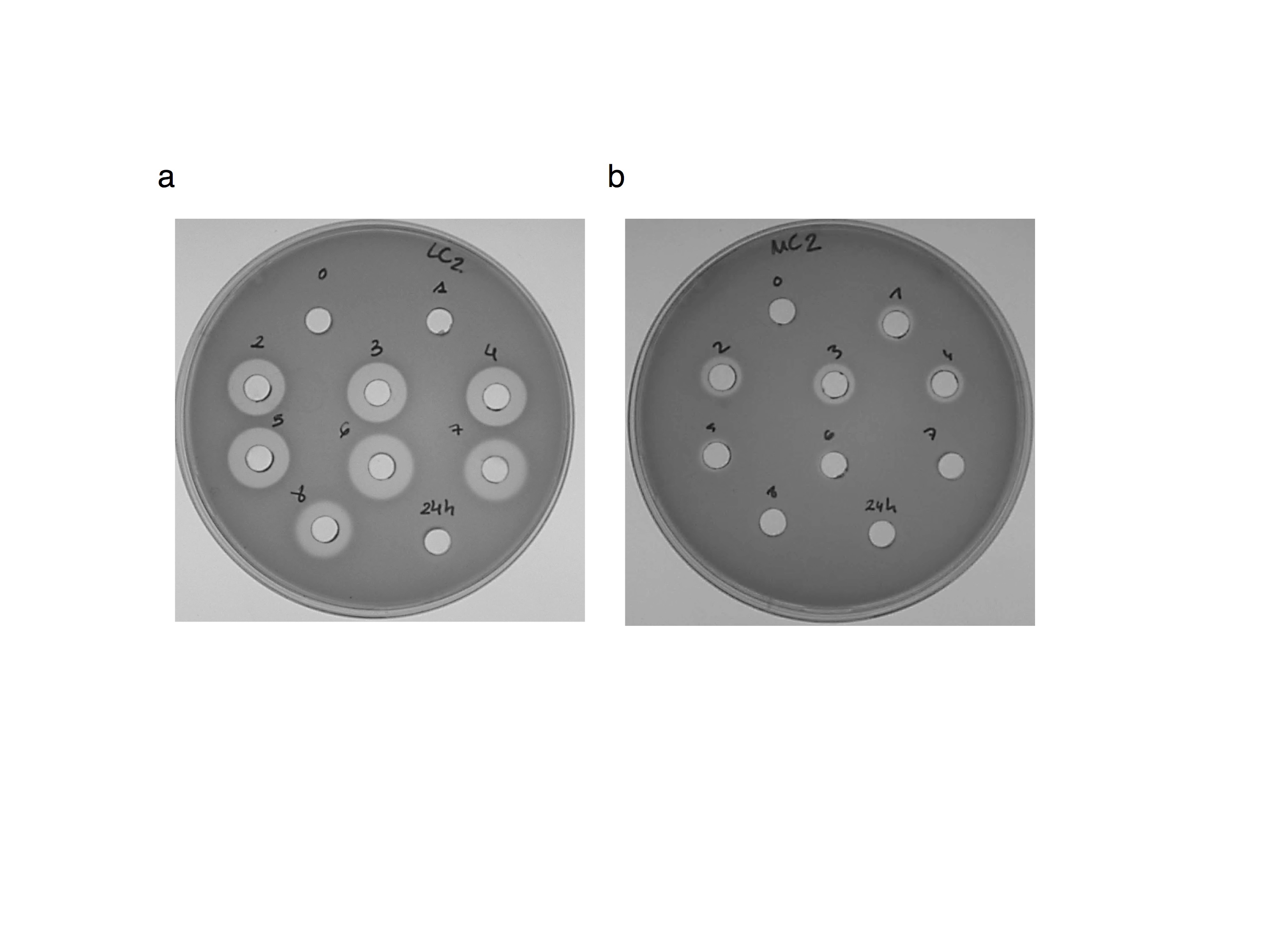
**

**Figure S3. Activity of bactofencin A against *Lb. bulgaricus* produced by *E.coli* Tuner^TM^ (DE3) cells carrying *bfn* vector.** Supernatants from cultures grown in the presence of a) glucose (low copy state, LC2) and b) arabinose (medium copy state, MC2) and 25 μM of IPTG for 0-8h and 24 h.

**Supplementary Table S1. Primers used in this** work

| **Primer name** | **Sequence 5’->3’** |
| --- | --- |
| **MCS F SphI** | gttcagcatgcgctagcccgggcgatcgcgacgtcggcgcgccgtacgcggccgcttaattaacctcaggcctaggacttg |
| **MCS R AvrII** | caagtcctaggcctgaggttaattaagcggccgcgtacggcgcgccgacgtcgcgatcgcccgggctagcgcatgctgaac |
| **bfn Fw1** | agaaggagatataagcatgttttttaattttatgaaaaaagtag |
| **bfn Rv1** | tcgcccgggctagcgcatgttactttaattgtaatgtacccttc |
| **pMPB1 bfnA Rv** | tcgcccgggctagcgcatgttagcaccaacgatacattcctg |
| **pMPB1 bfnAtail Rv** | ttttataccatcctaaacaattaataaaaaataaaacac |
| **pMPB1 bfnI Fw** | agaaggagatataagcatgtttagtttgacaccttatcaaaacc |
| **pMPB1 bfnI Rv** | tcgcccgggctagcgcatgttatctaaactttacaaaatctaaaatac |
| **pMPB1 bfnItail Rv** | attaaagatcctcctctataaaaaaagccagactcacgtc |
| **pMPB1 DLSL52 Fw2** | gtttaggatggtataaaatagctatttaaaaagtatacataatatg |
| **pMPB1 DLSL52 AvrII Rv** | gctcgagtgcggcctaggttaatttttcacaagtttataatac |
| **pMPB1 DLSL52 Rv** | tcgcccgggctagcgttaatttttcacaagtttataatac |
| **pMPB1 DLSL53 Fw2** | gaggaggatctttaatagctttggagatggaatttttgtac |
| **pMPB1 DLSL53 Fw3** | gtttaggatggtataaaaagctttggagatggaatttttgtac |
| **pMPB1 DLSL53 AvrII Rv** | gctcgagtgcggcctaggttactttaattgtaatgtacccttc |
| **pMPB1 DLSL53 Rv** | tcgcccgggctagcgttactttaattgtaatgtacccttc |
| **pMPB1 pedA Fw1** | agaaggagatataagcatgaaaaaaattgaaaaattaactg |
| **pMPB1 pedA Rv1** | gctcgagtgcggcctaggctagcatttatgattaccttg |
| **pMPB1 pedA Rv1int** | ctacaatatcccctttatcagtactagcatttatgattac |
| **pMPB1 pedB Fw1** | agaaggagatataagcatgaataagactaagtcggaac |
| **pMPB1 pedB Rv1** | gctcgagtgcggcctaggctattggctaggccacgtattg |
| **pMPB1 pedC Fw1int** | tactgataaaggggatattgtagttgtctaagaaattttg |
| **pMPB1 pedC Rv1** | gctcgagtgcggcctaggctactgattattgtaatcagcag |
| **pMPB1 pedD Fw1** | tcacctggttaatatggttttgtaaccaatgtaaaagg |
| **pMPB1 pedD Rv1** | gctcgagtgcggcctaggctattcttgattatgaattaacc |
| **pMPB1 pedAD Rv1** | caaaaccatattaaccaggtgactagcatttatgattacc |
| **pMPB1 pedBD Fw1** | ctgataaaggggatattgtagtcacctggttaatatggttttg |
| **pMPB1 pedBD Rv1** | ctacaatatcccctttatcagtactattggctaggccacg |

**Supplementary Table S2. Primers used to analyse the vectors by sequencing**

| **Primer name** | **Sequence 5’->3’** |
| --- | --- |
| **pMPB1 Fw1** | gagatataagcatgcgctagc |
| **pMPB1 Rv1** | tgccgtaaagcactaaatcgg |
| **T7** | taatacgactcactataggg |
| **bfn Fw2** | ggcataagttgggttacatgc |
| **bfn Fw3** | gatgaggtagagaaagcggttg |
| **bfn Rv3** | ctgcagcaccacagtcctcc |
| **bfn Rv4** | gtcgttttcccagaaccgctc |
| **bfn Fw5** | cgattctacctttgattatag |
| **pedA Fw12** | cattggtggtaaatactacg |
| **pedB Rv2** | ctattggctaggccacgtattg |
| **pedB Fw3** | gaataagactaagtcggaac |
| **pedC Rv4** | ccatattaaccaggtgactac |
| **pedC Fw5** | ggtctttcagcatggcttctc |
| **pedD Rv6** | gtcatcaagtgcggcaaataag |
| **pedD Fw7** | ccagattgcaattatcatagc |
| **pedD Rv8** | gatcagctttttgataagcc |
| **pedD Fw9** | gctcgttagttgtggttcc |
| **pedD Rv10** | gaacataattaatatattgg |
| **pedD Fw11** | cctagccaagttgctagttg |
